# Exercício Físico e MicroRNAs: Mecanismos Moleculares na Hipertensão e Infarto do Miocárdio

**DOI:** 10.36660/abc.20210538

**Published:** 2022-06-06

**Authors:** Alex Cleber Improta-Caria

**Affiliations:** 1 Universidade Federal da Bahia Programa de Pós-Graduação em Medicina e Saúde Salvador BA Brasil Programa de Pós-Graduação em Medicina e Saúde, Universidade Federal da Bahia, Salvador, BA – Brasil

**Keywords:** MicroRNAs/genética, Hipertensão, Infarto do Miocárdio, Exercício, Esforço Físico

## Introdução

Evidências científicas mostram que a prática regular de exercício físico (EF) é benéfica para diversos órgãos e sistemas do corpo humano, principalmente para o coração e sistema cardiovascular.^1^ Em ambos os sistemas, o EF aeróbico e de força promovem hipertrofia cardíaca fisiológica, respectivamente excêntrica e concêntrica, melhorando a função miocárdica.^2^

Além dos benefícios para o coração, o EF impacta os vasos sanguíneos através do estresse de cisalhamento e altera a função vascular em longo prazo, melhorando a função das células endoteliais e das células musculares lisas, gerando remodelação arterial e um potencial efeito antiaterogênico.^3^ Esses benefícios sobre o sistema cardiovascular ocorrem tanto em indivíduos saudáveis quanto em indivíduos com doenças cardiovasculares, como hipertensão arterial sistêmica (HAS)^4^ e infarto do miocárdio (IM),^5^ por exemplo.

No entanto, os mecanismos moleculares que governam esses benefícios induzidos pelo EF ainda não foram completamente elucidados, principalmente os mecanismos regulados por microRNAs (miRs), que são pequenos RNAs não codificantes que modulam o padrão de expressão gênica e de proteína em indivíduos saudáveis e naqueles com doenças cardiovasculares.^6^

Assim, o presente estudo tem como objetivo enfatizar a importância do EF na prevenção e tratamento da HAS e IM, bem como explicar o papel dos miRs induzidos pelo EF nestas condições patológicas.

### Hipertensão arterial sistêmica, miRs e EF

A HAS é uma doença multifatorial e está associada a fatores genéticos e fatores de risco modificáveis, como dieta hipercalórica e rica em sal, tabagismo, estresse, comportamento sedentário e inatividade física, sendo considerada fator de risco independente para IM.^7^ O EF, por sua vez, é extremamente benéfico para indivíduos com HAS, pois reduz os níveis pressóricos após o treinamento.^8^ Essa diminuição da pressão arterial deve-se, em parte, ao remodelamento arterial, atenuando a resistência vascular periférica e também devido à redução da atividade nervosa simpática.^8^ Entretanto, o papel dos miRs na redução da pressão arterial permanece incerto.

Poucos estudos demonstraram o papel regulador dos miRs na redução da pressão arterial. Em um estudo, os autores mostraram que o EF aeróbico reduziu a pressão arterial em ratos hipertensos ao reduzir a expressão do miR-16 que tem como alvo o gene do fator de crescimento endotelial vascular (VEGF), com consequente aumento da expressão do VEGF, melhorando a função endotelial e diminuição da expressão do miR-21, com consequente aumento do seu alvo, Bcl-2, atenuando a apoptose, demonstrando assim que o EF promoveu uma alteração nos fatores angiogênicos e apoptóticos, minimizando as anormalidades microvasculares e gerando revascularização periférica na HAS.^9^

Nesse contexto, também foi demonstrado que o EF aeróbico aumentou a expressão do miR-27a, reduzindo a expressão do seu alvo, o gene ACE, aumentou a expressão do miR-155, reduzindo a expressão do AT1R, e diminuiu a expressão do miR-153, aumentando a expressão do ACE2. Essas alterações moleculares induzidas pelo EF geraram alterações no fenótipo da artéria aorta em ratos hipertensos, como redução do peso e comprimento da aorta, redução da espessura da parede, atenuação da expressão de elastina e hidroxiprolina, com consequente melhora no relaxamento da aorta e da função endotelial, diminuindo a pressão arterial.^10^

Em outro estudo, o EF aeróbico aumentou a expressão de miR-145 com modulação da via de sinalização AKT, induzindo a alteração do fenótipo das células musculares lisas vasculares em ratos hipertensos, diminuindo a espessura da camada média, promovendo remodelamento arterial e reduzindo a pressão arterial sistólica e diastólica.^11^

Corroborando os estudos acima mencionados, outro estudo também mostrou que o EF reduziu a pressão arterial sistólica em ratos hipertensos, mas um aumento na expressão de miR-214 foi observado nesse estudo, exacerbando a disponibilidade de cálcio intracelular e o relaxamento de cardiomiócitos isolados.^12^

Assim, o EF é uma excelente ferramenta para modular a expressão de miRs e regular as vias de sinalização, induzindo alterações fenotípicas cardíacas e vasculares de longo prazo em ratos hipertensos; entretanto, esses experimentos ainda precisam ser feitos em humanos com HAS, para verificar se esses efeitos observados em estudos *in vivo* ocorrem em humanos.

### Infarto do miocárdio, miRs e EF

O IM é uma condição na qual o fluxo sanguíneo é reduzido em uma ou mais artérias coronárias, resultando em redução no fornecimento de oxigênio e nutrientes para alguns cardiomiócitos, com consequente morte dessas células. O IM é considerado uma das principais causas de morbidade e mortalidade em todo o mundo.^13^ Por outro lado, a prática regular de EF é importante para prevenir e tratar os indivíduos após IM, mas os mecanismos moleculares desses benefícios precisam ser melhor elucidados.

Em relação aos efeitos do EF na expressão de miRs em modelos animais pós-IM, o EF aeróbio aumentou a expressão de miR-29a, miR-29b e miR-29c, diminuindo a expressão dos genes COL1A1 e COL3A1, reduzindo o conteúdo de colágeno no miocárdio de ratos pós-IM quantificados pela concentração de hidroxiprolina, promovendo melhora na função cardíaca avaliada por ecocardiografia.^14^

**Figura 1:**
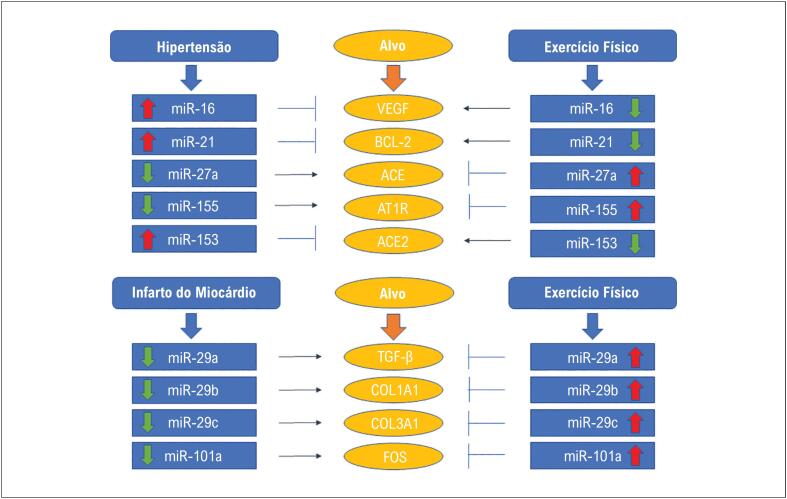
EF modulando miRs e alvos em HAS e IM.

Outro estudo também mostrou que o EF aeróbio exacerbou a expressão de miR-29a, inibindo a expressão de TGF-β e inativando sua via de sinalização, que é pró-fibrótica. Além do miR-29a, os autores também mostraram que o PE aumentou a expressão do miR-101a, que tem como alvo o gene FOS, reduzindo sua expressão e atenuando ainda mais a via do TGF-β. Essas alterações moleculares induzidas pelo EF resultaram em redução da fibrose intersticial miocárdica em ratos pós-IM^15^ (Figura 1).

Portanto, o EF tem um grande potencial para reduzir o perfil fibrótico cardíaco em ratos pós-IM através da modulação de miRs; entretanto, esses resultados também precisam ser elucidados em humanos, tanto a nível molecular quanto tecidual.

## Conclusões

Em conclusão, o EF é uma excelente estratégia para prevenir e tratar indivíduos com HAS e pós-IAM. Os miRs modulados por EF têm sido descritos como reguladores das vias de sinalização, induzindo modificação do fenótipo cardíaco e vascular em ratos hipertensos, promovendo redução da pressão arterial, hipertrofia cardíaca fisiológica e remodelação arterial, com melhora da função endotelial. Além disso, miRs modulados por EF também regularam as vias de sinalização associadas ao processo de fibrose cardíaca em ratos pós-IM, melhorando a função cardíaca. No entanto, esses efeitos benéficos dos miRs regulados por EF têm sido descritos em modelos animais, necessitando de ensaios clínicos para confirmar esses resultados obtidos *in vivo*, sendo essa uma nova linha de pesquisa promissora e desafiadora.
