# Supplementary material for: Exercício Físico e MicroRNAs: Mecanismos Moleculares na Hipertensão e Infarto do Miocárdio
Source: Arq Bras Cardiol. 2022 Jun 6;118(6):1147–9. [Article in Portuguese] doi: 10.36660/abc.20210538 (PMC9345150; doi:10.36660/abc.20210538)
Supplement: Translation

# Translation: Physical Exercise and MicroRNAs: Molecular Mechanisms in Hypertension and Myocardial Infarction

**Authors:** Alex Cleber Improta-Caria

**Affiliations:** 1 Salvador BA Brazil Programa de Pós-Graduação em Medicina e Saúde, Universidade Federal da Bahia, Salvador, BA – Brazil

**Keywords:** MicroRNAs/genetic, Hypertension, Myocardial Infarction, Exercise, Physical Exertion

## Introduction

Scientific evidence shows that the regular practice of physical exercise (PE) is beneficial for various organs and systems of the human body, mainly for the heart and cardiovascular system.^1^ In both systems, aerobic and strength PE promote physiological cardiac hypertrophy, respectively eccentric and concentric, improving myocardial function.^2^

In addition to the benefits for the heart, PE impacts blood vessels through shear stress and alters long-term vascular function, improving endothelial cell and smooth muscle cell function, generating arterial remodeling and a potential antiatherogenic effect.^3^ These benefits on the cardiovascular system occur in both healthy individuals and individuals with cardiovascular diseases, such as systemic arterial hypertension (SAH)^4^ and myocardial infarction (MI),^5^ for example.

However, the molecular mechanisms that govern these PE-induced benefits have not been completely elucidated, especially the mechanisms regulated by microRNAs (miRs), which are small non-coding RNAs that modulate the pattern of gene and protein expression in healthy individuals and those with cardiovascular diseases.^6^

Thus, the present study aims to emphasize the importance of PE in the prevention and treatment of SAH and MI, as well as explaining the role of PE-induced miRs in these pathological conditions.

### Systemic arterial hypertension, miRs and PE

SAH is a multifactorial disease and is associated with genetic factors and modifiable risk factors, such as a high-salt and high-calorie diet, smoking, stress, sedentary behavior and physical inactivity, being considered an independent risk factor for MI.^7^ PE in turn, is extremely beneficial for individuals with SAH because it reduces pressure levels after training.^8^ This decrease in blood pressure is due in part to arterial remodeling, attenuating peripheral vascular resistance and also due to reduced sympathetic nerve activity.^8^ However, the role of miRs in reducing blood pressure remains unclear.

Few studies have demonstrated the regulatory role of miRs to reduce blood pressure. In one study, the authors showed that aerobic PE lowered blood pressure in hypertensive rats by reducing the expression of miR-16 that targets the vascular endothelial growth factor (VEGF) gene, with a consequent increase in VEGF expression, improving endothelial function and decrease in miR-21 expression, with a consequent increase in its target, the Bcl-2, attenuating apoptosis, demonstrating that PE promoted an alteration in angiogenic and apoptotic factors, minimizing microvascular abnormalities, and generating peripheral revascularization in SAH.^9^

In this context, it was also shown that aerobic PE increased the expression of miR-27a, decreasing the expression of its target, the ACE gene, increased the expression of miR-155 reducing the expression of the AT1R and decreased the expression of miR-153, increasing the expression of ACE2. These molecular changes induced by PE, generated changes in the phenotype of the aorta artery in hypertensive rats, such as reduced aortic weight and length, decreased wall thickness, attenuation of elastin and hydroxyproline expression, with consequent improvement in the relaxation of the aorta and endothelial function, decreasing blood pressure.^10^

In another study, aerobic PE increased the expression of miR-145 with modulation of the AKT signaling pathway, inducing the phenotype change of vascular smooth muscle cells in hypertensive rats, decreasing the medial layer thickness, promoting arterial remodeling and decreasing systolic and diastolic blood pressure.^11^

Corroborating the abovementioned studies, another study also showed that PE reduced systolic blood pressure in hypertensive rats, but an increase in miR-214 expression was observed in this study, exacerbating the availability of intracellular calcium and the relaxation of isolated cardiomyocytes.^12^

Thus, PE is an excellent tool to modulate the expression of miRs and regulate signaling pathways, inducing long-term cardiac and vascular phenotypic changes in hypertensive rats; however, these experiments still need to be performed in human beings with SAH, to ascertain whether these effects observed in *in vivo* studies occur in humans.

### Myocardial infarction, miRs and PE

MI is a condition in which blood flow is reduced in one or more coronary arteries, resulting in a reduction in the supply of oxygen and nutrients to some cardiomyocytes, with consequent death of these cells. MI is considered one of the main causes of morbidity and mortality worldwide.^13^ On the other hand, regular PE practice is important to prevent and treat the individuals after an MI, but the molecular mechanisms of these benefits need to be further elucidated.

Regarding the effects of PE on the expression of miRs in post-MI in animal models, aerobic PE increased the expression of miR-29a, miR-29b and miR-29c, decreasing the expression of COL1A1 and COL3A1 genes, reducing the collagen content in the myocardium of post-MI rats quantified by the concentration of hydroxyproline, promoting improvement in cardiac function assessed by echocardiography.^14^

Another study also showed that aerobic PE exacerbated the expression of miR-29a, inhibiting the expression of TGF-β, inactivating its signaling pathway, which is pro-fibrotic. In addition to miR-29a, the authors also showed that PE increased the expression of miR-101a, which targets the FOS gene, decreasing its expression and further attenuating the TGF-β pathway. These PE-induced molecular changes resulted in reduced myocardial interstitial fibrosis in rats after MI^15^ (Figure 1).

**Figure 1:** PE modulating miRs and targets in SAH and MI.

Therefore, PE has a great potential to reduce the cardiac fibrotic profile in post-MI rats through the modulation of miRs; however, these outcomes also need to be elucidated in humans, both at the molecular and tissue level.

## Conclusions

Finally, PE is an excellent strategy to prevent and treat individuals with SAH and post-MI. PE-modulated miRs have been described as regulators of signaling pathways inducing modification of the cardiac and vascular phenotype in hypertensive rats, promoting blood pressure reduction, physiological cardiac hypertrophy and arterial remodeling, with improved endothelial function. Furthermore, PE-modulated miRs also regulated signaling pathways associated with the cardiac fibrosis process in post-MI rats, improving cardiac function. However, these beneficial effects of PE-regulated miRs have been described in animal models, requiring clinical trials to confirm these results obtained *in vivo*, being a promising and challenging new line of research.
